# Surgical Management of Radicular Cyst With the Application of a Natural Platelet Concentrate: A Case Report

**DOI:** 10.7759/cureus.33992

**Published:** 2023-01-20

**Authors:** Lokhasudhan Govindaraju, Delphine P Antony, Pradeep S

**Affiliations:** 1 Conservative Dentistry and Endodontics, Sri Ramachandra Institute of Higher Education and Research, Chennai, IND; 2 Conservative Dentistry and Endodontics, Saveetha Dental College-Saveetha Institute of Medical and Technical Sciences, Chennai, IND

**Keywords:** radicular cyst, platelet rich fibrin, retrograde filling, pulp necrosis, mineral trioxide aggregate, plasma rich fibrin

## Abstract

Traumatic dental injuries usually involve the dentoalveolar region, and they readily affect the teeth and their surrounding soft and hard tissues. The common sequelae of traumatic dental injuries lead to pulpal necrosis and apical periodontitis along with cystic changes. The current case report describes the surgical management of a radicular cyst in the periapical region of maxillary incisors and highlights the efficacy of natural platelet concentrate [platelet-rich fibrin (PRF)] used for postoperative healing. A 38-year-old male patient presented to the department with pain and mild swelling in the upper front tooth region. On radiographic examination, a radiolucent periapical lesion was evident in relation to the right maxillary central and lateral incisor. In the maxillary anterior region, root canal therapy was performed, followed by periapical surgery and retrograde filling with mineral trioxide aggregate (MTA), and PRF was placed in the surgical site to initiate the healing at a faster rate. The patient was recalled for follow-ups after 12 weeks, 24 weeks, and 36 weeks; he was found to be asymptomatic, and significant periapical healing was observed in the radiograph with almost adequate bone formation.

## Introduction

Cyst-like lesions are always associated with infections in the intra-radicular and extra-radicular regions [1]. Such lesions do not show any symptoms of pain but demonstrate a progressive increase in size on the radiograph [2]. The surgical approach is often preferred when there is an invariable change in the peri-radicular tissue that could not be treated by nonsurgical management. Platelet-rich fibrin (PRF) is associated with the concept of obtaining a rich layer of platelet concentrate. It accumulates platelets and the released cytokines in a fibrin clot. The significant step in this process is the quantification of platelet cytokine in the PRF, as these soluble molecules are key inflammation and healing mediators [3]. PRF is a natural structure derived from blood associated with immune and platelet concentrate that collects all the constituents of blood to enable rapid wound healing [4,5]. PRF contains multiple growth factors like platelet-derived growth factor (PDGF), transforming growth factor β1 (TGF β1), insulin-like growth factor (IGF), vascular endothelial growth factor (VEGF), etc., and exhibits several vital properties like cell attachment, cell migration, cell proliferation, and cell differentiation [6]. Also, it was previously considered to be a potent biomaterial for the regeneration of the pulp-dentin complex [7]. PRF has been shown to be an ideal interpositional and healing biomaterial. It acts as an effective and strong barrier between desired and undesired cells and as an interpositional material by stopping the early invagination of undesired cells. When used as a healing biomaterial, it promotes wound closure rapidly and the healing of mucous membranes by releasing growth factors and fibrin dressing [5]. Unlike platelet-rich plasma (PRP), PRF does not require biochemical manipulation of the blood. The preparation of PRF involves a simplified process and is also cost-effective. To promote the conversion of fibrinogen to fibrin, PRF eliminates the redundant process of adding bovine thrombin in PRP.

## Case presentation

**Figure 1:**
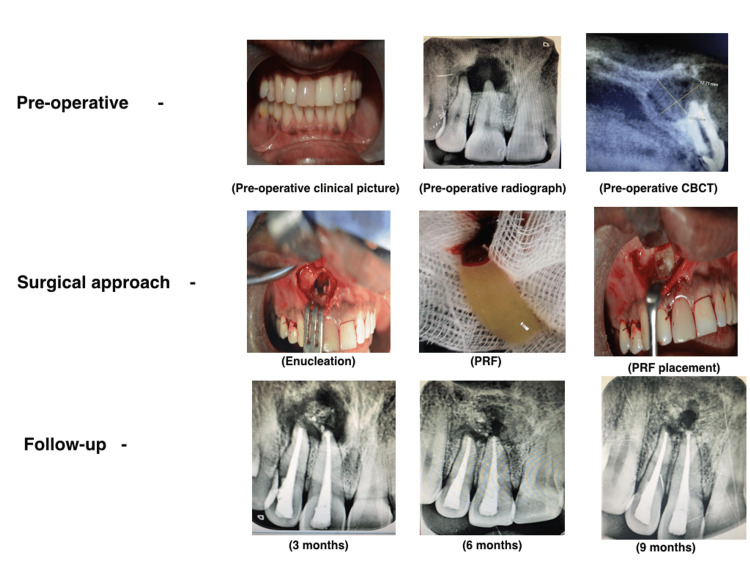
Preoperative, intraoperative, and follow-up pictures of periapical lesion management CBCT: cone-beam computed tomography; PRF: platelet-rich fibrin

A 38-year-old male patient reported to the Department of Conservative and Endodontics with the chief complaint of pain, discoloration, and mild swelling in the palatal aspect for one year; he also had a history of trauma 15 years prior. Clinical examination revealed a discolored maxillary right central incisor (11), which was asymptomatic. A pulp sensibility test revealed no response. Radiographic examination showed periapical radiolucency in relation to 11 and 12, and cone beam CT (CBCT) showed a supernumerary tooth above the right maxillary premolars. Based on these findings, the treatment plan was made in such a way that both procedures would be done at the same time. From the sagittal view, the extent of the lesion was 12 mm in diameter labio-palatally (Figure 1).

Endodontic treatment protocol

Access opening was done in relation to 11 and 12 and the working length was measured using an apex locator, which was then confirmed with a radiograph. The cleaning and shaping were done using K-file. The master apical file was #50K-file and the step-back technique was done up to #80K-file; 5.25% sodium hypochlorite was used as irrigant followed by 17% EDTA gel (Anabond Endoprep-Rc) during biomechanical preparation, and saline was used as a final irrigant. Calcium hydroxide (RC Cal, Prime Dental, Mumbai, India) was used as an intracanal medicament, placed inside the prepared root canal twice with an interval of one week, but the patient was still symptomatic. Periapical surgery was planned and so the obturation was completed using gutta-percha points by lateral condensation technique using AH Plus® sealer.

Preparation of PRF

Around 10 ml of venous blood was extracted from the patient at the time of surgery and collected in two sterile vacutainer tubes of 5 ml each without anticoagulant. Those tubes were processed in a tabletop centrifugation machine at 3000 rpm continuously for almost 10 minutes. As a final product, it forms the following layers: starting from the bottom, a red lower layer containing red blood cells; the middle layer containing the fibrin clot; and an upper straw-colored layer containing cellular plasma. The middle layer containing the fibrin clot (PRF) was collected by dividing the red blood cells and plasma. More importantly, the junction layer of cellular plasma and the fibrin was carefully preserved as it contains high platelet concentrates.

Surgical treatment protocol

The surgical procedure was done under general anesthesia since palatal and labial approaches were planned as well as the extraction of the supernumerary tooth and enucleation of the cyst. The infiltration of local anesthesia was done with adrenalin at the maxillary anterior region. A full-thickness mucoperiosteal flap was raised and, initially, the extraction of the supernumerary tooth was done by the palatal approach followed by cyst enucleation on the labial aspect. A biopsy was taken for pathological analysis. Apicoectomy was done followed by root-end preparation, and the retrograde filling was given with MTA; 10 ml of blood was taken prior to the surgery with the patient's consent. The blood was transferred to the test tube and centrifuged at 3000 rpm for 10 minutes to obtain clear PRF. The PRF was placed in the cystic cavity and suturing was done (Figure 1).

The biopsy taken during the surgery was examined, and a radicular cyst was confirmed. Later, the patient was reviewed at follow-ups after three, six, and nine months. No symptoms such as pain, inflammation, or discomfort were observed during the review period. Routine intraoral examinations and professional plaque control were done during the follow-up visits (Figure 1).

## Discussion

The development of a periapical cyst is a gradual and continuous process. Epithelial cell rests of Malassez are stimulated by an inflammatory process, and cystic fluid made up of cholesterol develops immediately around the apex. Cystic expansion occurs from cystic fluid or it can become infected [8]. Lateral root displacement as well as tooth mobility is pathognomonic of cysts. Cysts constitute about 15% of all periapical lesions, and radicular cysts are the most common among them and account for nearly half of all periapical lesions. Among odontogenic cysts, the periapical cyst is the most common type with an incidence rate of 52.3-70.7%, followed by the dentigerous cyst with 16.6-21.3% and odontogenic keratocysts with an incidence of about 5.4-17.4% [9]. The treatment option is based on the consideration of several factors such as the origin and extent of the lesion, its relationship to vital structures, clinical features, systemic conditions, and patient cooperation [10]. The treatment of these cysts is still a matter of dispute and controversy. Many professionals choose endodontic therapy as a conservative method for smaller lesions. In our case, root canal treatment was performed over multiple visits with provisional calcium hydroxide medicament. The application of intracanal dressings over multiple visits is important for reducing microbial levels when compared to mechanical preparation in cases of chronic periapical lesions. Calcium hydroxide dressings help to penetrate areas that are untouched by instruments or irrigation solutions, such as dentinal tubules and ramifications. Calcium hydroxide, due to its hygroscopic properties, was shown to have high clinical efficacy in reducing the exudate. Studies have shown that a minimum of two weeks is necessary for the placement of calcium hydroxide medicament for its antimicrobial activity [11]. However, in large periapical lesions, endodontic treatment will not allow the complete eradication of the bacteria, and hence either marsupialization/decompression or even enucleation should be done [12].

However, surgery would be required if it is a real cyst, as it is autonomous and hence does not respond well to endodontic treatment. AH Plus®, an epoxy resin-based sealer, was used as it has more antimicrobial activity than other sealers against endodontic pathogens. PRF, in addition to its capacity to promote healing, has proven effective in reducing postoperative hematoma, due to its favorable sealing ability with fibrin adhesive [13]. The effectiveness of PRF application completely relies on the amount of time taken to collect the blood and transfer it to the centrifuge immediately. However, the blood gets coagulated as it comes into contact with the tubes without anticoagulant, and it takes a minimum amount of time for centrifugation to acquire concentrated fibrinogen in the middle and upper parts of the tube [14]. In order to obtain a clinically usable PRF, prompt manipulation is required and it is the only feasible method. If the duration for collecting blood and centrifuging is longer, it will become ineffective. It will result in the diffuse polymerization of the fibrin acquired in the tube and only a minimal amount of fibrinogen without any consistency will be obtained [15]. MTA is often the material of choice as it has better biocompatibility, bacteriostatic activity, and favorable sealing ability as a root-end filling. MTA has been shown to have clinical and radiographic success in inducing apical root-end closure [16-18]. A significantly higher rate of healing (about 92%) was observed after six months with PRF [19] in the study by Thanikasalam et al.

## Conclusions

Surgical management of radicular cysts with the application of PRF membrane significantly releases the key growth factors slowly and sustainably, which makes the membrane stimulate the environment for a significant period of time during its remodeling. The clinical success of PRF confirms its efficacy as a healing biomaterial during periapical surgery. Hence, clinicians should understand the benefits of PRF and use it while performing surgeries, which will provide a cost-effective option for the patients.
